# The skin autofluorescence may help to select patients with Type 2 diabetes candidates for screening to revascularization procedures

**DOI:** 10.1186/s12933-024-02121-5

**Published:** 2024-01-13

**Authors:** Fadi Alkhami, Gauthier Borderie, Ninon Foussard, Alice Larroumet, Laurence Blanco, Marie-Amélie Barbet-Massin, Amandine Ferriere, Claire Ducos, Kamel Mohammedi, Sami Fawaz, Thierry Couffinhal, Vincent Rigalleau

**Affiliations:** 1 Endocrinology-Diabetology-Nutrition and Cardiology, Bordeaux CHU and University, 33000 Bordeaux, France; 2 Endocrinology-Nutrition, CHU Bordeaux, Hospital Haut-Lévêque, Avenue de Magellan, 33604 Pessac, France

**Keywords:** Advanced glycation end-products, Skin autofluorescence, Type 2 diabetes, Revascularization

## Abstract

Chen et al. recently related the skin autofluorescence (SAF) of Advanced Glycation End-products to subclinical cardiovascular disease in the 3001 participants from the general population (Rotterdam study), with a particularly close relationship for the 413 subjects with diabetes. Because conventional vascular risk factors do not capture the risk in diabetes very well, this relationship may help to select high-risk individuals for the screening of silent myocardial ischemia, which has yet to prove its benefit in randomized controlled trials. Among 477 patients with uncontrolled and/or complicated Type 2 Diabetes, we measured the SAF ten years ago, and we registered new revascularizations during a 54-months follow-up. The patients with SAF > 2.6 Arbitrary units (AUs), the median population value, experienced more revascularizations of the coronary (17/24) and lower-limb arteries (13/17) than patients with a lower SAF, adjusted for age, sex, diabetes duration, vascular complications, and smoking habits: HR 2.17 (95% CI: 1.05–4.48), p = 0.035. The SAF has already been reported to predict cardiovascular events in three cohorts of people with diabetes. We suggest that its measurement may help to improve the performance of the screening before vascular explorations and revascularizations.

We were interested in the recent article from Jinluan Chen et al., who related the skin autofluorescence (SAF) to markers of subclinical cardiovascular disease in the Rotterdam study [1]: carotid plaques and intima-media thickness, coronary artery calcifications (CAC), and pulse wave velocity. Besides the scientific interest in the role of Advanced Glycation End-products in cardiovascular disease in the general population, their results may have practical implications for subjects with diabetes.

As mentioned by the authors, the relationship between SAF and subclinical cardiovascular disease was especially close for their 413/3001 participants with diabetes. In Type 2 Diabetes (T2D), the intima-media thickness [2], the pulse wave velocity [3], and the CAC scores [4] are predictive of later cardiovascular events, whereas conventional risk factors do not well capture this risk. They may help to identify high-risk individuals with T2D for the screening of myocardial ischemia, as proposed for the CAC score in a position article of the French Societies of Cardiology and Diabetology: screen patients with CAC scores > 400 Arbitrary units (AUs) [5].

Myocardial ischemia is often silent in people with T2D, arguing for this screening. However, randomized controlled trials that tested it have not yet detected a benefit [6]. The selection of high-risk subjects seems therefore critical, and the relationship to subclinical cardiovascular disease makes SAF an interesting candidate as its measurement is simple and non-invasive. As an important objective of the screening is to select subjects who could benefit from a revascularization to prevent a cardiovascular event, the article from Chen prompted us to test whether the SAF related to later revascularizations in our cohort of subjects with T2D, that recently allowed us to show that the SAF predicted Diabetic Foot Ulcers [7].

The characteristics of the 477 subjects are presented in the Table 1. They were hospitalized in our diabetology unit from 2009 to 2017 for uncontrolled and/or complicated T2D. All were interviewed, had a clinical examination, and blood and urine samples. To participate in the study, which was approved by the local ethics committee, all the subjects gave their informed consent to the measurement of SAF, the anonymized collection of variables and outcomes from their medical records, and their analysis. As expected, due to our hospital setting, our patients were poorly controlled (HbA1c 8.7 ± 1.8% or 72 ± 14.9 mmol/mol), with high rates of vascular complications.

**Table 1:** Baseline characteristics of the patients with later revascularizations vs patients without

	Whole population	Indemn of revascularization	New revascularization	p
N	477	436	41	
Sex (% men)	56.8%	55.4%	71.4%	0.051
Age (years)	62 ± 9	61 ± 9	64 ± 9	0.062
Duration of diabetes (years)	14 ± 10	14 ± 9	17 ± 10	0.050
HbA1c (%)	8.7 ± 1.8%	8.7 ± 1.8%	8.6 ± 1.7%	0.826
BMI (kg/m2)	32.7 ± 6.1	32.6 ± 6.2	33.4 ± 5.5	0.439
Triglycerides (mg/dL, median, IQR)	158 (112–226)	159 (113–223)	154 (106–290)	0.810
HDL-cholesterol (mg/dL)	44 ± 13	44 ± 14	42 ± 10	0.309
LDL-cholesterol (mg/dL)	105 ± 43	105 ± 43	99 ± 38	0.422
Treated by a statin (%)	65.2%	64.1%	76.2%	0.122
Arterial hypertension (%)	64.8%	63.9%	73.8%	0.238
Smoking (%)	23.1%	21.4%	40.5%	0.011
Albumin Excretion Rate (mg/24H, median, IQR)	15 (4–62)	14 (4–47)	53 (8–360)	0.001
Estimated GFR (mL/min/1.73m2)	82 ± 25	83 ± 24	72 ± 28	0.006
Macroangiopathy (%)	31.4%	27.8%	69.0%	0.000
Retinopathy (%)	25.8%	24.4%	40.5%	0.027
Diabetic kidney disease (%)	43.8%	41.4%	69.0%	0.001
Skin autofluorescence (AU)	2.67 ± 0.64	2.64 ± 0.62	3.00 ± 0.73	0.000

Forty-one revascularizations were registered during the 54 ± 27 months of follow-up: 24 coronary and 17 lower-limbs. The revascularized patients differed from others for higher rates of vascular complications at baseline, whereas no difference for conventional risk factors (arterial hypertension, dyslipidemia) was significant, except for more frequent smoking habits. The SAF were higher for later revascularized patients than non-revascularized patients (p < 0.001).

The Fig. 1 depicts revascularization-free survival curves according to a SAF higher vs lower than the median value (2.6 AUs), that differed (Log-Rank: p < 0.001). They similarly differed for coronary (Log-Rank: p = 0.005) and lower-limbs (Log-Rank: p = 0.007) revascularizations. For the 319 subjects without macroangiopathy (defined as previous myocardial infarction, stroke, peripheral revascularization) at baseline, 5 revascularizations were performed and all were in subjects with SAF > 2.6 AUs (Log-Rank: p = 0.008). By Cox regression analysis, a SAF > 2.6 AUs was related to later revascularizations: HR 2.17 (95% CI: 1.05–4.48) p = 0.035, adjusted for age, sex, diabetes duration, smoking, and diabetic vascular complications defined as macroangiopathy, retinopathy and Diabetic Kidney Disease (glomerular filtration rate < 60 ml/min/1.73m^2^ and/or albumin excretion rate 30 > mg/24H).

**Fig. 1:**
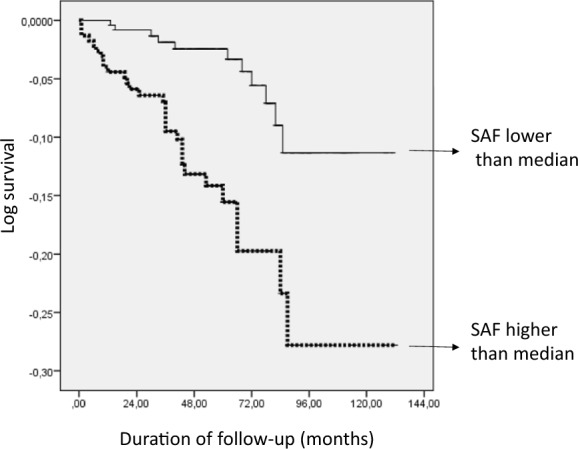
Revascularization-free survival curves in 477 subjects with T2D, according to their SAF higher vas below the median (2.6 AUs). Log-Rank: p < 0.000

The higher rate of revascularizations among T2D patients with high SAF is well-accorded with its relationship to subclinical cardiovascular disease as reported by Chen. Higher risk of cardiovascular events related to SAF were already reported in three cohort studies [8–10], but they did not specifically address revascularization procedures. Advanced glycation endproducts (AGEs) score measured at the fingertip in patients with cardiovascular disease have been associated with major adverse cardiovascular and cerebrovascular events [11]. Elevated SAF was recently associated with subclinical atherosclerosis in coronary and carotid arteries independently of conventional risk factors [12]. Moreover, both advanced glycation expressed by higher skin autofluorescence or soluble receptor for AGEs (sRAGE) and impaired microvascular reactivity are involved in the pathogenesis of vascular complications in diabetes [13]. We hope that the Rotterdam study team will follow their participants during the next years, to confirm whether the SAF may help to select subjects for further coronary and lower-limbs vascular explorations, and revascularizations if necessary.

## Data Availability

No datasets were generated or analysed during the current study.

## Abbreviations

AU: Arbitrary Units
CAC: Coronary Artery Calcifications
SAF: Skin autofluorescence
T2D: Type 2 Diabetes

## References

[1] Chen J, Arshi B, Waqas K, Lu T, Bos D, Ikram A (2023). Advanced glycation end products measured by skin autofluorescence and subclinical cardiovascular disease: the Rotterdam Study. Cardiovasc Diabetol.

[2] Yoshida M, Mita T, Yamamoto R, Shimizu T, Ikeda F, Ohmura C (2012). Combination of the Framingham risk score and carotid intima-media thickness improves the prediction of cardiovascular events in patients with type 2 diabetes. Diabetes Care.

[3] Kim HL, Jeon WK, Joh HS, Lim WH, Seo JB, Kim SH (2022). Brachial-ankle pulse wave velocity as a predictor of long-term cardiovascular events in 2174 subjects with type 2 diabetes mellitus: a retrospective cohort study. Medicine (Baltimore).

[4] Kramer CK, Zinman B, Gross JL, Canani LH, Rodrigues TC, Azevedo MJ (2013). Coronary artery calcium score prediction of all cause mortality and cardiovascular events in people with type 2 diabetes: systematic review and meta-analysis. BMJ.

[5] Valensi P, Henry P, Boccara F, Cosson E, Prevost G, Emmerich J (2021). Risk stratification and screening for coronary artery disease in asymptomatic patients with diabetes mellitus: position paper of the French Society of Cardiology and the French-speaking Society of Diabetology. Arch Cardiovasc Dis.

[6] Mohammedi K, Préaubert N, Cariou T, Rigalleau V, Foussard N, Piazza L (2021). Cost-effectiveness of screening of coronary artery disease in patients with type 2 DIABetes at a very high cardiovascular risk (SCADIAB study) rational and design. Cardiovasc Diabetol.

[7] Borderie G, Foussard N, Larroumet A, Blanco L, Barbet-Massin MA, Ducos C (2023). The skin autofluorescence of advanced glycation end-products relates to the development of foot ulcers in type 2 diabetes: a longitudinal observational study. J Diabetes Complications.

[8] Lutgers HL, Gerrits EG, Graaff R, Links TP, Sluiter WJ, Gans RO (2009). Skin autofluorescence provides additional information to the UK Prospective Diabetes Study (UKPDS) risk score for the estimation of cardiovascular prognosis in type 2 diabetes mellitus. Diabetologia.

[9] Jin Q, Lau ESH, Luk AOY, Ozaki R, Chow EYK, Cheng F (2021). Skin autofluorescence is associated with higher risk of cardiovascular events in Chinese adults with type 2 diabetes: a prospective cohort study from the Hong Kong Diabetes Biobank. J Diabetes Complications.

[10] Boersma HE, van Waateringe RP, van der Klauw MM, Graaff R, Paterson AD, Smit AJ (2021). Skin autofluorescence predicts new cardiovascular disease and mortality in people with type 2 diabetes. BMC Endocr Disord.

[11] Hirai T, Fujiyoshi K, Yamada S, Matsumoto T, Kikuchi J, Ishida K (2023). Association between fingertip-measured advanced glycation end products and cardiovascular events in outpatients with cardiovascular disease. Cardiovasc Diabetol.

[12] Pan J, Bao X, Gonçalves I, Jujić A, Engström G (2022). Skin autofluorescence, a measure of tissue accumulation of advanced glycation end products, is associated with subclinical atherosclerosis in coronary and carotid arteries. Atherosclerosis.

[13] Škrha J, Horová E, Šoupal J, Valeriánová A, Malík J, Prázný M (2022). Skin autofluorescence corresponds to microvascular reactivity in diabetes mellitus. J Diabetes Complications.

